# An assessment of the state of conservation planning in Europe

**DOI:** 10.1098/rstb.2023.0015

**Published:** 2024-05-27

**Authors:** Martin Jung, Diogo Alagador, Melissa Chapman, Virgilio Hermoso, Heini Kujala, Louise O'Connor, Rafaela Schinegger, Peter H. Verburg, Piero Visconti

**Affiliations:** ^1^ Biodiversity, Ecology and Conservation Research Group, International Institute for Applied Systems Analysis (IIASA), Schlosspark 1, Laxenburg, 2361, Austria; ^2^ Biodiversity Chair, MED: Mediterranean Institute for Agriculture, Environment and Development, 7006-554, University of Evora, Portugal; ^3^ Department of Environmental Science, Policy, and Management, University of California Berkeley, Berkeley, CA 94720, USA; ^4^ Department of Plant Biology and Ecology, University of Sevilla, 41012, Seville, Spain; ^5^ Finnish Museum of Natural History, 00100 Helsinki, Finland; ^6^ Laboratoire d'Ecologie Alpine, Université Grenoble Alpes, Université Savoie Mont Blanc, CNRS, LECA, Grenoble, F-38000 Grenoble, France; ^7^ University of Natural Resources and Life Sciences Vienna, 1180 Vienna, Austria; ^8^ VU University Amsterdam, 1081 HV Amsterdam, The Netherlands; ^9^ Swiss Federal Institute WSL, CH-8903 Birmensdorf, Switzerland

**Keywords:** biodiversity conservation, spatial optimization, prioritization, ‘30 × 30’, decision theory, stakeholder

## Abstract

Expanding and managing current habitat and species protection measures is at the heart of the European biodiversity strategy. A structured approach is needed to gain insights into such issues is systematic conservation planning, which uses techniques from decision theory to identify places and actions that contribute most effectively to policy objectives given a set of constraints. Yet culturally and historically determined European landscapes make the implementation of any conservation plans challenging, requiring an analysis of synergies and trade-offs before implementation. In this work, we review the scientific literature for evidence of previous conservation planning approaches, highlighting recent advances and success stories. We find that the conceptual characteristics of European conservation planning studies likely reduced their potential in contributing to better-informed decisions. We outline pathways towards improving the uptake of decision theory and multi-criteria conservation planning at various scales, particularly highlighting the need for (a) open data and intuitive tools, (b) the integration of biodiversity-focused conservation planning with multiple objectives, (c) accounting of dynamic ecological processes and functions, and (d) better facilitation of entry-points and co-design practices of conservation planning scenarios with stakeholders. By adopting and improving these practices, European conservation planning might become more actionable and adaptable towards implementable policy outcomes.

This article is part of the theme issue ‘Ecological novelty and planetary stewardship: biodiversity dynamics in a transforming biosphere’.

## 1. Introduction

There is an urgent need to halt the decline of biodiversity in the EU, and the ecosystem services it supports. Despite important past efforts to preserve biodiversity, such as the Birds and Habitats Directives or Water Framework Directive, there has been insufficient progress towards halting biodiversity decline [1–4]. For this reason, the European Union (EU) has committed to an ambitious biodiversity recovery plan supported by the Biodiversity Strategy for 2030, the Green Deal [5] and backed by the Global Biodiversity Framework [6]. These policy advances aim to set biodiversity in a recovery path and move towards sustainable development, focusing on the restoration of degraded habitats, protecting undisturbed lands, extending the network of protected areas and improving the effectiveness of management, governance and funding in this coming decade. Where and how these ambitioned goals are to be achieved, however, depends on the strategic allocation of conservation measures under limited and uncertain budgets [7] and an overall strong competition for land resources by multiple sectors. Given these challenges there is thus a need for robust decision-making into where and what to achieve with conservation and restoration actions in space and time.

European land- and seascapes have been shaped by a long history of intense anthropic use [8]. Many European landscapes can be classified as cultural landscapes that originate from distinct historical management processes, landscape structures and the constant evolution of human values [9,10]. Undisturbed natural areas are scarce [11,12] and most European land- and seascapes are firmly embedded in production systems that provide agricultural and fishery products, timber and other recreational functions [13]. At the same time, the historical management legacies of these unique cultural landscapes have, over time, shaped biodiversity and created unique habitats that are dependent on low-intensity management [14]. Managing such land- and seascapes in a way that is compatible with historic low-intensity practices and cultural practices could help to conserve those biodiversity aspects, which are often also in greatest need of conservation efforts [4]. This also highlights the necessity that European conservation measures consider context-specific aspects of cultural and management practices to maximize synergies and trade-offs between human use and biodiversity [14].

Europe has a long history of planning directives and policies with regard to nature conservation. The Natura 2000 network of internationally designated protected areas has been a tremendous success story, constituting the largest coordinated network of areas for managing biodiversity in the world. It covers 18.5% of land area in the EU and 9% of the sea [15] and it is complemented by nationally designated areas (national park, regional reserves and other national designations) covering an additional 7.9% of land, as of April 2023 (see the official EEA dashboard at https://www.eea.europa.eu/data-and-maps/dashboards/natura-2000-barometer).

In the marine realm, in addition to national and international designations following the EU Nature Directives and other national and international commitments (e.g. Convention on Biological Diversity protected area targets) a strong support for marine spatial planning has come from the EU marine strategy framework directive [16] which made marine spatial planning compulsory for all its member states [17]. Furthermore, the EU Water Framework Directive [18] and related River Basin Management Planning constitute a novel baseline for catchment-based planning and integrative water resources management across Europe.

The EU policy biodiversity strategy targets include a commitment to expand nature conservation through the means of area-based conservation targets (e.g. share of 30% protected areas of land and sea, of which one-third are under strict protection). While area-based percentage area targets have been criticized [19] for directing efforts towards the means (protection) rather than the ends (conserving habitats, species and ecological processes), a strategic implementation of these targets through the means of spatial planning can help ensure their meaningful contribution towards the overarching objectives of the Strategy (i.e. biodiversity conservation).

Planning for nature conservation can be conducted at various scales, each with its own purpose and way of contributing to decision-making processes and achieving overarching policy objectives. European- or regional-scale efforts are most effective at highlighting areas of broad conservation importance or identifying cross-border and transboundary collaboration opportunities [20,21]. National level planning, in contrast, is more suited to take into account country-specific datasets or accounting processes that might not be available or comparable at broader scales [22]. Ultimately, the implementation of any place-based conservation measures is usually done in local contexts, and under consultation and negotiation with relevant local and regional stakeholders and planning authorities. Although various planning approaches are widely applied globally, no comprehensive overview on their methodological and spatial application nor specific purpose exists for a European context.

Spatial planning can be achieved through the means of systematic conservation planning (SCP), which is a decision-theoretical framework that can help bridge the gap between politically driven area targets and the stated ambition of conserving biodiversity by identifying a set of areas requiring conservation management that satisfy a series of conservation objectives (e.g. for species, ecosystems or ecological processes). It leverages tools from decision theory to identify optimal management strategies in the face of uncertainty and multiple, often competing, objectives [23,24]. SCP approaches may not be exclusively applied for the identification of new protected areas, for which they have played a key role in recent decades [25]. They also can support the identification of optimal management actions [26], enable representation and rank priorities in terms of their importance or value for conservation management [27,28] and evaluate different scenarios given synergies and trade-offs of multiple objectives [27,29–31]. SCP approaches further allow the flexible integration of quantitative and qualitative evidence, including perspectives and visions of stakeholders [32–34]. Although SCP approaches are highly promising to guide strategic implementation and decision-making, it is unclear to what extent they have been applied across European landscapes and seascapes.

SCP can be conducted in a range of different ways and with a wide variety of analytical frameworks [35–37]. Frameworks and planning tools get more complex as they try to integrate more ecological complexity, socio-economic constraints and processes to inform the implementation of policies. For example, current rates of changes in climate and land use fundamentally affect the efficiency and role of protected areas, and new advances in SCP have enabled the better accounting of dynamics such as species distribution shifts in future protected area designations [38–40]. Other methodological developments have enabled a better integration of uncertainties [41], costs [42], connectivity [43,44], responses to management scenarios [45] and multi-objective optimizations [27,31,46,47], all of which add more nuance and realism to the resulting plan, potentially contributing to successful adoption of results. Ultimately, any implementation is dependent on the involvement of stakeholders and efforts have been taken to incorporate their visions, feedback and concerns at various stages of SCP exercises [33,35]. It is however not yet clear to what extent these advances have proliferated into SCP or other planning approaches.

In the recent past, a few studies systematically reviewed and mapped SCP studies and similar approaches globally [48–51]. However, to our knowledge, no such assessment has been conducted specifically for the European context. Given the timeliness of EU policies and the strong legacy of SCP application across Europe, we specifically review the scientific literature to assess where and how SCP and related conservation planning approaches have been applied in a European context. We explore the properties and indicators of complexity applied in the available literature and evaluate the uptake of these studies in subsequent scientific and policy documents. By also drawing on previous reviews at a global scale, we discuss prospects, directions and gaps in conservation planning applications in Europe and highlight opportunities for more integration across objectives, models and realms, emphasizing the critical role that SCP in particular will play in reaching European conservation policy targets across scales.

## 2. Methods

We conducted a structured review of existing scientific literature, covering references that applied SCP and other related approaches in a European context (including the UK, Norway and all Balkan states in the process of joining the European Union). We purposefully excluded global studies and set our focus on articles from the European geographical context, but recognize that a rich source of the literature that has applied such approaches on other continents also exists [50]. We primarily focused on literature with a conservation or restoration planning aim, including biodiversity conservation issues and gap analyses. Although the focus of the review was on SCP approaches (see search term in electronic supplementary materials), other approaches for identifying where to act or what to do were also captured. We did not specifically consider literature focusing on other fields (e.g. landscape- or urban planning), but explicitly record if and how land-use aspects have been considered in the analysed literature (table 1). To identify relevant literature, we used the Scopus search engine. We recognize that other search engines might yield slightly different results, but do not know of any systematic bias that would affect the broad scale patterns observed in this analysis. We also acknowledge other review guidelines that might increase repeatability in the future (http://prisma-statement.org/). The query was run on the 23rd of September 2022 and resulted in an initial 1459 articles for screening. A full list of the terms passed to the search engine can be found in the electronic supplementary material.

**Table 1.** Depiction of variables, types, and the rationale behind the collection in the review.

variable name	unit/values	rationale
extent	local | national | regional | Europe	Qualitative depiction of the extent of planning from small to larger scale.
region	country name	The name of the European country in which the study was conducted. Enter multiple names or ‘Europe’ if study was conducted across multiple regions. Broad regions if the countries are not explicitly stated (Mediterranean)
locality	free text	Any further information with regards to the region discussed in free text.
realm	terrestrial | freshwater | marine |cross-realm	Realm of the planning exercise.
ecosystem specificity	type	Covers whether specific to certain ‘ecosystems’ such as prioritization within coastal regions or forests. Single text description.
period	contemporary | future | both	Temporal period considered in the planning scenario. With 'contemporary', we refer to any dataset collected prior to the conducted analysis. Future or dynamic planning approaches usually include or consider variables or constraints beyond the 2030 period. Analysis would consider both the contemporary state and future conditions (e.g. future climatic refugia).
purpose of planning	type (single categorical term).	Broad categorization of the goal of the planning exercise, being for instance identification of priority areas for management implementation (e.g. placement of protected areas, usage zones, restoration), specific actions (e.g. eradication of invasive species), or representation (e.g. coverage of features) or identification of synergistic effects (e.g. minimization of costs across objectives).
policy relevance	name of relevant policies or ‘none’; in the case of a specific concrete case-study, enter ‘case study’	Whether the work is explicitly trying to address objectives of a policy as opposed to being curiosity- or methodologically-driven (e.g. demonstration of a new method using a particular EU case study).
algorithm approach	method	Listing the tool or approach used for planning, i.e. Marxan
biodiversity type	species | ecosystems | Nature Contributions to People (NCP) | other	What types of biodiversity data were considered in the work and at what level was it included.
multiple objectives or management and land-use constraints	none|multi-objective |constraints| costs| other	Indication of whether the planning exercise has addressed multiple objectives (besides biodiversity) or accounted for or incorporated constraints related to anthropogenic production, constraints and/or land-use practices.
connectivity	boundary | structural | functional | none	Was connectivity in any way considered in the planning. Available options include boundary connectivity (avoid clumping), structural connectivity through habitat features and functional connectivity through species dispersal and trait features.
costs	yes/no	Were costs related to opportunity or land purchases considered?
stakeholder involvement	yes/no	Were proposed planning inputs or outcomes communicated, informed or co-designed together with relevant stakeholders?
number of features	number	How many features were considered in the process.

We screened the title and abstract of each of the resulting articles for suitability for inclusion in the review, and removed all studies that were broadly irrelevant, i.e. unlikely to have applied any planning approaches in a biodiversity-relevant context, or were conducted at an irrelevant extent (globally, or outside of European Member states/the European continent). This yielded a total of 356 studies across terrestrial, freshwater and marine realms, which we then assessed in more detail by extracting a set of key variables and criteria relevant for this review (table 1). Finally, we also complemented these studies with other sources of scientific literature about conservation planning beknown to the authors. This was done in order to specifically include studies that were not indexed by Scopus, making use of a ‘snowballing’ system, where any added known study was screened for their cited references and assessed for further literature not yet considered.

All found European studies were then assessed for their fulfilment of different criteria of planning complexity (table 1). These criteria are whether a study accounted for connectivity, considered current/future conditions and competing land-uses, and had indicated policy relevance or involved stakeholders. For each identified study in our final dataset, we extracted information on the ‘uptake’ in the scientific and policy literature. As a generic proxy for 'uptake’, we make use of the number of citations in scientific and policy documents, realizing that this number can only give a conservative estimate of the true relevance of a work. We used the ‘rscopus’ package to extract this information for each article with a Digital Object Identifier [52] and here mainly relied on the PlumX citation metrics, which provide insights into by whom (social media, scientific, policy documents) and when articles are cited. See the Data accessibility section for access to the full list of studies considered. We furthermore provide an online interactive website to navigate through the outputs of the analysis (https://martin-jung.github.io/Review_EuropeanConservationPlanning).

Finally, we used the parameters extracted from the literature (table 1) and interrogated the data in terms of who cited them using a series of Bayesian regression models. In the case of citations, we used zero-inflated Poisson distributed Bayesian regression models to estimate the conditional effect of certain parameters on the relative rate of citations. We used a zero-inflated link function to account for the articles that have never been cited and furthermore included a temporal random intercept to account for year-to-year differences, given that older studies are more likely to have accumulated citations over time. Bernoulli distributed regressions were used for all other analyses, where the aim was to test for differences in the mean. Analyses were conducted using the ‘brms’ package [53,54].

## 3. Review findings

**Figure 1.**
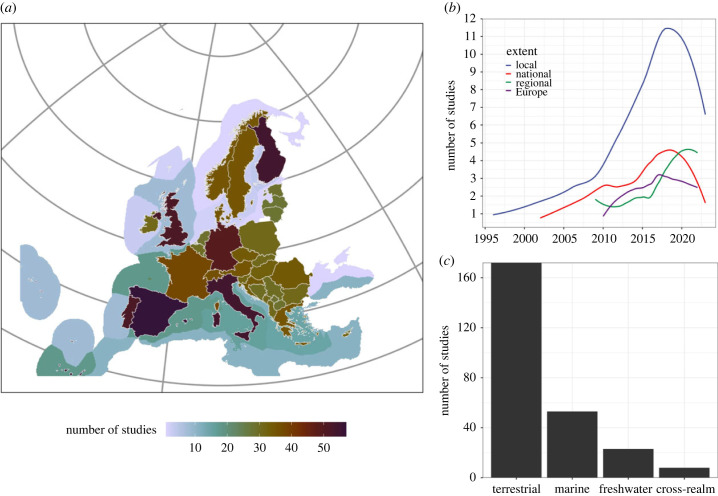
Spatial and temporal patterns of conservation planning studies in Europe. Shown are the spatial distribution (*a*), the temporal trend of studies separated by spatial extent (*b*), and the number of studies by realm (*c*).

In total, we found 266 suitable studies covering 40 individual countries, or broader regional and European extents across the period from 1996 to 2023 (figure 1*b*). European studies using SCP and related approaches were mostly conducted on land (67% of all studies), followed by marine studies (20%). The most represented countries are Spain (10.7% of all studies), the UK (10%), Portugal (9.1%) and Finland (9.1%, figure 1*a*). Studies predominantly covered local scales (54%), with the fewest number of studies being conducted at the European scale (11.3%, figure 1*b*). Most studies aimed to either identify priority areas (40.3%) or investigate representation gaps and sufficiency (30%) of existing protection measures. The most applied methodological approaches were using heuristics (see also electronic supplementary material, table S1), with most of the studies using standalone software such as Marxan (31% of all studies), followed by Zonation (20%) or ranking and scoring approaches (19.7%). The remaining studies used exact algorithms such as Integer programming (19.4%); with other approaches such as multi-criteria analyses or machine learning accounting for the remaining 29.9%. Most planning studies considered multiple ecosystem types in terms of priorities for conservation areas or actions (62%). Close to half of all studies (46%) used features that only considered species in their work, ranging from 1 to over 4447 species (electronic supplementary material, figure S1), although an increasing number of studies also considered ecosystem services (10%, electronic supplementary material, figure S1) or multiple feature types together (24%).

**Figure 2.**
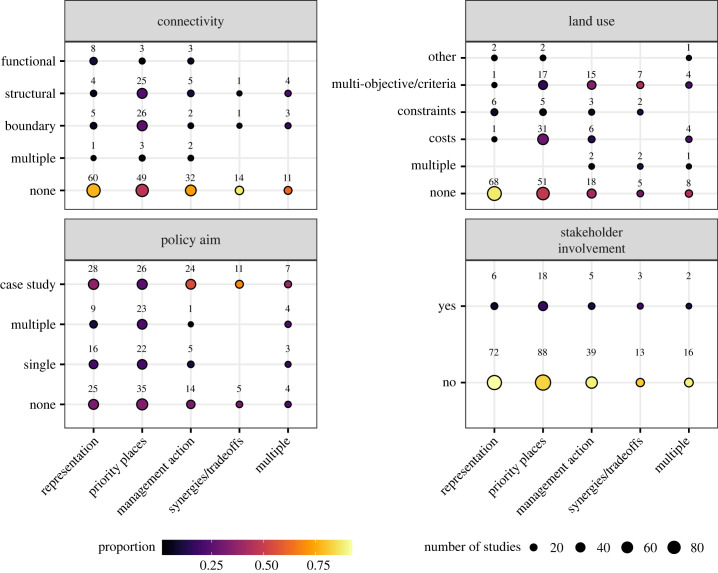
Overview of the properties of the studies identified in the review. Shown are factors related to connectivity, whether a study accounted for land use, and if the study aimed at a particular policy objective and whether stakeholders have been involved in any capacity in the conceptualization of the study. The bottom axes separate between levels of these factors related to the study aim. The sizes of the points indicate the numbers of studies, while the colours show the proportions of all studies for the study aim (bottom axes).

Interestingly, not even one of the assessed studies of European extent fulfilled all criteria for an adequate accounting of the complexities of spatial planning (table 1 and figure 2). An overwhelming proportion of studies (87%) considered only contemporary data on biodiversity, land use, climate and other factors. Similarly, most studies focused on entire landscapes (62%) instead of making specific assessments, for example, forest conservation priorities (8.3%). Only 37% of all studies accounted somehow for connectivity in the prioritization, with the most common approach being neighbourhood constraints, which penalized the selection of isolated areas for conservation. Even fewer studies considered future states of their features (12.9%), i.e. using future distributions of species or habitats, or anticipated costs.

Remarkably, very few studies (11.1%) involved stakeholders in the conceptualization or execution of their study, although 68% of all studies aimed to be relevant for or to influence one or more policies. Notably, not a single conservation planning study at European scale considered the views of stakeholders (electronic supplementary material, figure S2). Conservation priorities often compete with alternative land- or water uses. Yet only 54.5% of all studies somehow accounted for these constraints, most commonly in the form of opportunity costs.

Citations of scientific and policy documents can serve as a coarse proxy of their uptake by the respective communities. On average, any given planning study was cited about 27 times (median: 17, range: 0–162) in scientific documents, 1.4 times (median: 0, range: 0–21) in policy documents and 22 times in other outreach channels such as social media, blog posts or news articles (range: 0–434). While on average, a study is cited about 2.9 times for every year after it has been published, it can take an average up to 4.4 years before a study is first cited in any policy document. We also find that that the probability that a scientific study is cited by policy documents decreases every year as increasing numbers of scientific studies are published (*β* = −0.05, d.f. = 261, *p* = 0.03). We found no correlation between the number of social media mentions and the number of citations in scientific (*r* = −0.06, d.f. = 261, *p* = 0.34) or policy documents (*r* = 0.02, d.f. = 261, *p* = 0.79). We did however find that articles that are cited more frequently by scientific documents also tended to be cited more often in policy documents (*r* = 0.45, d.f. = 261, *p* < 0.001).

**Figure 3.**
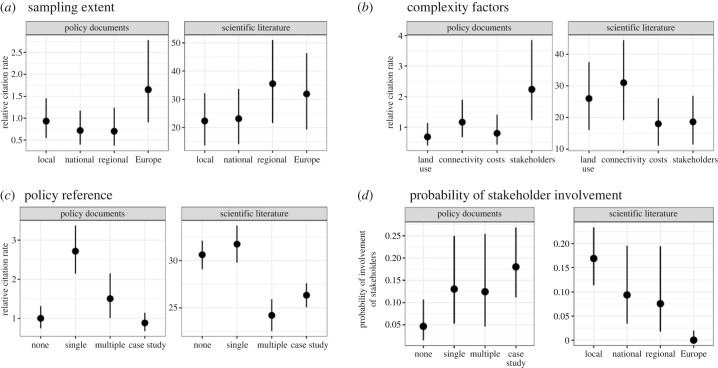
The results of a Bayesian regression model assessing the difference in the number of citations in policy or scientific literature, or the probability of stakeholder involvement, differ depending on certain properties of the reviewed studies.

The number of citations by scientific and policy documents differed according to the properties of the respective studies (figure 3). We found that studies at European extent were more often cited than comparable studies at local extent by both policy documents ($\lambda_{\mathrm{Europe}}$ = 1.7) and scientific ($\lambda_{\mathrm{Europe}}$ = 26) documents published in the same year (figure 3). While studies that accounted for aspects of connectivity (*λ* = 31) or land use (*λ* = 26) were on average cited more frequently by scientific documents than comparable studies, the best determinant of higher citation rates by policy documents was the involvement of stakeholders in the study (*λ* = 2.24, figure 3). Studies that considered the views of stakeholders usually had case study-specific policy objectives and were of smaller extent (figure 3). Whether or not studies have made reference to none or some of the policy contexts resulted in small differences in scientific citations (figure 3, *λ*_none_ = 28, *λ*_single_ = 32). However, studies that referred to a single policy context were more often cited in policy documents (*λ* = 2.8), although there was little difference in the uptake by scientific documents with regards to whether a study had or had not referred to any policy contexts (figure 3). Overall, these results highlight that the magnitude of uptake of SCP and related approaches differs among scientific and policy audiences with regard to study scale and complexity.

## 4. Discussion

Expansion of areas under effective conservation management is a key aspect in European biodiversity policies. However, given the limited financial resources, trade-offs between preservation of biodiversity and other competing human objectives, there is a need to prioritize efforts. In this work, we reviewed the scientific literature on SCP and related approaches in Europe to understand where and how planning for places and actions has been applied across scales and realms. Clear patterns of predominant practices emerged, and crucially we found that not a single study accounted for all aspects of planning complexity that might be preferable in a well-designated spatial plan of conservation priorities (table 1). Very few studies (13%) accounted for future conditions in some form, which is a large oversight given that climate change is expected to shift the distribution of most European species and habitats [38,55] and conservation management is expected to contrast projected land-use change, where it would have the highest negative impacts on biodiversity [56]. Nevertheless, we show that many SCP studies, specifically those referring to specific policy contexts, are cited in policy documents, highlighting the relevance of prioritization studies as scientific evidence and decision support. Unfortunately, in Europe, which SCP studies have resulted in the designation of new spatially targeted conservation areas or improved management and restoration actions is not known or officially documented.

Implementing the EU Biodiversity strategy for 2030 requires engaging diverse stakeholders, including the general public, about how biodiversity management should be conducted within and outside protected areas [57]. We find that—regardless of the method of consultation, scale or purpose of planning—stakeholder inputs are rarely considered in European planning studies (figure 2). This result aligns with the findings of global reviews of integrated land-sea planning studies, which found that most did not involve or consult stakeholders in any form [58]. Further, our results suggest that studies considering the views of stakeholders were on average more often cited by policy documents than scientific literature (figure 3*d*), which could hint at a disconnect between the relevance of conservation prioritization studies for scientific outlets and policy making. Although the benefits and necessities in addressing and integrating drivers of biodiversity decline across scales and extents are well identified [59,60], is also notable that not a single study at European scales engaged with stakeholders and only a few at national or regional scales, which might further enforce the perception that conservation planning can be seen as a ‘top–down’ approach or ‘black-box’ driven by science.

Terrestrial studies by far dominated the scientific literature, whereas freshwater and cross-realm planning approaches, e.g. those that consider terrestrial as well as freshwater and/or marine systems [21,35,37], have rarely been conducted (figure 1). This constitutes a problematic issue, as there is increasing evidence that drivers behind biodiversity pressures are interlinked across realms [59]. We thus amplify previous calls to step up implementations and proof-of-concept case studies of cross-realm European planning to adequately design and implement catchment plans that can contribute to jointly halting the decline of freshwater and terrestrial biodiversity [1].

Overall, 55.4% of investigated studies consider some type of socio-economic cost or constraint in their planning (figure 2). And additionally, studies that incorporated multiple objectives criteria received on average 10% more scientific citations than those studies that did not, and they make up less than half of all studies that incorporated non-biological factors into their planning exercises. This aligns with previous reviews of conservation planning studies that highlighted a general focus on biological rather than socio-economic patterns and processes [48]. In a European context that can be an issue as the heterogeneity and governance of different land systems necessitate planning concepts that ideally fulfil multiple functions for nature, economy and society [14,61]. Integrated planning has been highlighted as key to address the multiple drivers of biodiversity decline and to maximize synergies across policy goals and realms [1,5,6,62,63]. Clearly, there is a need to further mainstream the consideration of multiple objectives in SCP, especially in working landscapes and for including nature's contributions to people.

### (a) Perspective on recent advances

Our review has shown that a wide range of approaches and tools have been applied in European planning studies and among them SCP remains the best-suited approach to prioritize and evaluate potential conservation outcomes and explicitly address conflicts and trade-offs.

#### (i) Novel tools and ease of access

SCP software continues to be developed and expanded in terms of complexity [25]. Recent developments include spatially optimal zoning [43,64], next-generation ranking algorithms for spatial prioritization [65], integer programming for planning for places and actions [66–68], restoration of specific landscapes patterns [69] or the use of reinforcement learning for identifying conservation priorities [70] or management actions [71]. Recent studies have proposed new objective functions that allow the achievement of multiple targets linearly [27,68] or by identifying more compact solutions with core areas to benefit species sensitive to edge effects [72]. Each of these approaches comes with their own promises, benefits and caveats (see electronic supplementary material, table S1) and there is no tool that is universally regarded as the most appropriate by all end-users.

Nevertheless, simple scoring methods remain pervasive in planning processes across scales (figure 2), often driven by the demand of stakeholders for transparent and understandable processes. This is worrying, given that these have been known to be imprecise and potentially misleading in identifying priorities for places or actions [73]. Most likely, there remains a lack of easily useable tools particularly for non-academics or those unfamiliar with developing analytical code, as well as a lack of capacity building to strengthen the skillset with existing tools. The move towards cloud-based prioritization software such as the Marxan Planning Platform (MaPP, https://marxansolutions.org/marxanmapp/) will hopefully also contribute towards reducing entry barriers for analysts with less technical training.

#### (ii) Towards integrated spatial planning

We highlight several methodological developments in SCP, for which we see a promise in their application in the context of spatial planning at the European extent (table 2). The integration of conservation actions within human-dominated landscapes makes conservation challenging, as any actions to restrict use and access will directly affect the users of these landscapes or displace other types of land use [86]. An answer could be integrated planning solutions, that balance competing demands between preservation of biodiversity, provision of ecosystem services and economic or cultural demands towards land and seascapes [31,62,87,88]. Such applications of SCP hold great promise in identifying priorities that integrate across policy sectors (horizontal) and scales (vertical). For example, in the context of the European Biodiversity Strategy it is necessary to ensure that management for conservation is effective, especially considering limited resources, and that it ideally maximizes benefits across varying policy objectives such as biodiversity and climate mitigation and adaptation goals [55,89].

**Table 2.** Recent conceptual and methodological innovations and developments in SCP, with exemplary studies for their application.

innovation	concept	key reference examples
planning for actions	Implicit consideration of what to do in a given context, for example by identifying optimal priorities to allocate or improve management actions for threat abatement.	targeted priorities for actions [26,67,74]
dynamic conservation planning	Explicit accounting of multiple temporal timesteps to identify priorities for spatial management zones or their actions.	ensuring resilience to future change [38,75]
		scheduling of management actions in the light of changing pressures [71,76]
formulations of robust biological informed targets	Current practices of setting feature targets in prioritizations are often naïve or abstract. New advances are being made in improving the formulation of targets to improve their biological realism	Targets informed by RedList criteria [27,77], Favourable reference values [78], indicators of landscape metrics [69]
integrated and joint planning across sectors	Integration of features and targets from different sectors into a single prioritization, thus allowing the balancing of synergies and trade-offs with biodiversity and other demands, for example, through weights.	Integrated planning for both biodiversity and land use targets [31,47,79–81]
		Joint planning balancing multiple objectives [27,82]
		Consideration of NCPs in SCP [27,36,83]
co-design with stakeholders	Involve those actors affected or benefiting by the implementation of the conservation priorities in the design and execution of the analytical process	Guidelines and examples for increased effectiveness [33], *co-design of planning with stakeholders* [79,84]
connectivity	Directly incorporate connectivity in the design of current or future reserve networks.	Incorporating ecological connectivity in SCP [43,44,85]

However, the data or required information are not always available for developing a spatial plan, and with greater complexity this increases the risk of incorrectly prioritizing areas or actions due to poor data or modelling assumptions [90]. For example, it has been found that planning solutions are particularly sensitive to aggregated constraints, e.g. those acting at the level of a single planning unit or over the whole area of interest [91]. The complex dynamics and socio-cultural factors (i.e. sense of place and ownership, land tenure) governing European working landscapes highlight the importance of considering non-biological factors in spatial plans. Yet, the critical decision of which socio-economic data are ‘good enough’ to include highlights issues of data harmonization and provenance. Overall, there seems an urgent need to better align social, economic and ecological objectives in spatial planning.

Similarly, most conservation planning approaches try to reduce planning complexity by using static ‘proxy’ variables for threats to biodiversity or level of intactness, such as aggregated ‘human-footprint’ indices. This lack of differentiation of separate pressures to biodiversity, and accounting for their specific impacts, impede the identification of appropriate management actions to abate these threats, and also come with the invalid assumptions that pressures have additive impacts [92]. Future integrated planning studies should attempt to better account for such idiosyncrasies by proposing ways to manage different types of land- and seascapes, ideally through linkages with domain-specific data and knowledge.

#### (iii) Dynamics and process-based planning

To ensure that SCP is fit for purpose to tackle the complexity of conservation problems, more conceptual work on expanding and testing common problem formulations for the selection of areas is needed. Current SCP approaches do not yet comprehensively evaluate the impact of future changes or account for dynamics of natural and socio-economic systems (figure 2; electronic supplementary material, table S1). Here a better incorporation of connectivity within the problem formulation could help to identify solutions that are more robust to future changes [43,44,85]. For example, the identification of adaptive dispersal corridors can help species populations to persist in the light of climate change and for future range expansion [38,93]. Further, novel approaches such as reinforcement learning bring some promises, for example, for spatial planning over highly dynamic problems or policy horizons [71]. At the same time, this also comes with the cost of interpretability because resulting planning solutions cannot be easily explained by analysts (typical ‘black box’) and the influence of data or model uncertainties cannot be comprehended. Clearly, there is a need to better understand uncertainties in parameter choices and how they influence resulting solutions and communicate them, especially towards stakeholders and less-trained analysts.

#### (iv) Adequacy of scale-specific planning

There is also an increasing realization that SCP needs to integrate across scales [60]. For example, while many management problems for protected areas might only effectively be addressed at local scale (figure 1), influencing pressures such as climate change or governance regulations and constraints to national biodiversity strategies occur beyond the local scale [94]. There is a need to identify practical ways forward regarding how bottom–up and top–down objectives can be integrated in spatial planning. Plausible scenarios for evaluating trade-offs, as well as a well-designed theory of change underpinning any proposed place or action-based implementation, will likely help to improve acceptance and policy impact of SCP. Supporting the implementation of SCP along all implementation steps in commonly used frameworks [37] would likely support a more effective implementation.

Moving forward, we also recommend that evidence and results from SCP studies should be more readily accessible across different scales and realms, and some such efforts have been spearheaded elsewhere in Gabon or globally [17,50,95]. One idea could be to build a European-wide reference database highlighting different SCP frameworks, case-studies and tools, by also providing the evidence behind these works (e.g. data, maps and indicators used for evaluation) in a transparent and digestible way. Similar databases already exist in the context of the European Marine Spatial Planning directive (Directive 2014/89/EU, https://maritime-spatial-planning.ec.europa.eu/msp-practice/database), but not for either terrestrial or freshwater systems. A European hub of SCP frameworks and evidence could help to improve cohesiveness and consciousness for conservation issues, particularly for cross-border or cross-realm challenges, and may contribute to a widespread adoption of SCP.

## 5. Conclusion

Our review on the current state of SCP in European contexts highlights the varying levels of complexity—or lack thereof—in how conservation decisions are determined. In a mission-driven discipline like conservation, scholarly work should strive to be useful and close to implementation. Policy relevance can be achieved through addressing many of the complexity factors outlined above, such as the following: (a) by incorporating stakeholder visions and preferences in SCP. The fact that stakeholders are rarely consulted in scientifically oriented SCP studies might hint at one of the symptoms explaining why protected areas are often of low efficiency, particularly if conflicting objectives and initial ‘buy-in’ by stakeholders are ignored; (b) by more comprehensively integrating multiple objectives and the socio-economic aspects that matter. Spatial plans for conservation in European multi-functional landscapes cannot be realized without considering the trade-offs with such management actions; (c) by considering robust policy contexts and targets for the biodiversity attributes that matter, rather than ‘proxy’ variables with little prospect of implementation or actionable advice; (d) by expanding and developing the set of openly available data and tools and providing decision makers with capacity-building opportunities and training to expand the use of SCP as standard.

SCP can contribute more than just maps. It can estimate the sufficiency of management plans, identify synergies and trade-offs or evaluate different planning scenarios in terms of their benefits. Addressing current and future conservation challenges can only happen through widespread adoption of best practices and use of evidence, with scientists and practitioners jointly contributing to this process in a concerted way. We thus urge European scientists to thus make further efforts to increase the relevance of their planning work.

## Data Availability

The datasets supporting this article have been uploaded as part of the electronic supplementary material and also from the Zenodo repository: doi:10.5281/zenodo.8104715 [96]. The analysis code used to create the figures and statistics are accessible from the GitHub repository: https://github.com/Martin-Jung/Review_EuropeanConservationPlanning [97]. The data are provided in electronic supplementary material [98].
